# Supplementary material for: Experimental structures of antibody/MHC-I complexes reveal details of epitopes overlooked by computational prediction
Source: bioRxiv. 2023 Dec 4:2023.12.01.569627. Preprint. [Version 1] doi: 10.1101/2023.12.01.569627 (PMC10723347; doi:10.1101/2023.12.01.569627)
Supplement: Supplement 1 [file media-1.pdf]

SUPPLEMENTARY MATERIALS

Experimental structures of antibody/MHC-I complexes reveal details of epitopes overlooked by computational prediction

Lisa F. Boyd, Jiansheng Jiang, Javeed Ahmad, Kannan Natarajan, and David H. Margulies

Molecular Biology Section, Laboratory of Immune System Biology, National Institute of Allergy and Infectious Diseases, National Institutes of Health, Bethesda MD, 20892-1892

SUPPLEMENTARY FIGURE 1.

SUPPLEMENTARY FIGURE 2.

SUPPLEMENTARY FIGURE 3.

SUPPLEMENTARY FIGURE 4.

SUPPLEMENTARY FIGURE 5.

SUPPLEMENTARY TABLE 1.

SUPPLEMENTARY TABLE 2.

SUPPLEMENTARY TABLE 3.

SUPPLEMENTARY TABLE 4.

Supplementary Fig. 1

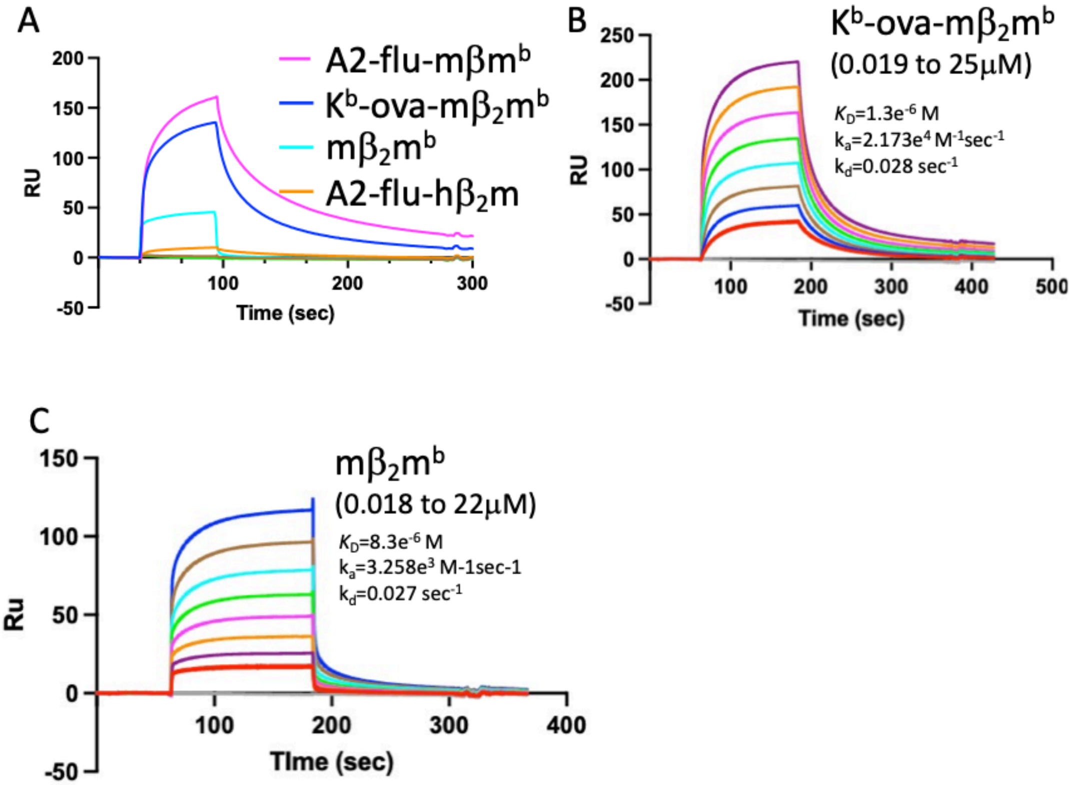

Supplementary Figure 1. S19.8 binds MHC-I molecules complexed with mouse β_2m^b but not with human β_2m. (A) SPR analysis was performed as described in Methods using a surface coupled with S19.8 (1100 RU), using preparations of pMHC consisting of: HLA-A*02:01/flu peptide/human β_2m or mouse β_2m^b, H2-K^b/ova peptide (SIINFEKL)/human β_2m or mouse β_2m^b, or free human β_2m or mouse β_2m^b at 1 uM. Baseline tracings reveal the lack of binding of free human β_2m (green) or of H2-K^b-ova-hβ_2m. (B) Graded concentrations (from 0.019 to 25 uM) of H2-K^b-SIINFEKL-β_2m^b were offered to S19.8. (C) Graded concentrations (from 0.18 to 22 uM) of mouse β_2m^b were offered to the S19.8 surface. Curves were fit globally to a single site kinetics

model using BiaEval 3.0. Indicated parameters are representative of three independent experiments with different preparations of S19.8.

Supplementary Fig.2

A

Complex	X-ray model	AF-M model	MHC-I (A+B+P)	A (α-chain)	B (β_2m)	P (pep)	Loop	MHC-I α1α2	MHC-I α3
34-5-8/D^d	8TQ8	8TQ8-AF	1.814	1.330	2.690	2.574	---	1.259	1.456
34-2-12/D^d	8TQ7	8TQ7-AF	1.360	1.369	1.278	2.101	---	1.272	1.534
28-14-8/D^b	8TQA	8TQA-AF	1.793	1.931	1.331	1.859	5.428	2.099	1.558
S19.8/D^d/ β_2m^b	8TQ9	8TQ9-AF	1.703	1.732	1.422	3.168	5.164	1.664	1.855

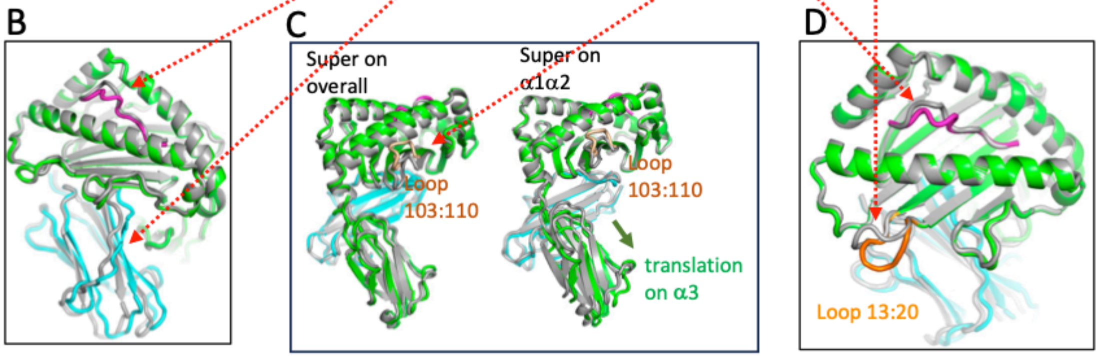

Supplementary Figure 2. Comparison of X-ray and AF-M models of pMHC in different complexes. The indicated Fab/MHC-I complexes were predicted by AF-M as described in the Methods and compared with the indicated refined crystallographic models. (A) Comparisons (reported as Å) were performed as all-atom RMSD using CNS 1.3 as described in Materials and Methods. All three chains (A+B+P), each protein chain alone (α, H chain; β_2m L chain), or individual domain units were compared as indicated. The hinge angle defined by α1α2 vs α3, was also calculated. (B) Superposition of H2-D^d from 8TQ8 as compared with the pMHC from the AF-M best_model for H2-D^d from the Fab34-5-8 complex. (C) Superposition of H2-D^b from 8TQA as compared with the pMHC from the AF-M best_model for H2-D^b from the Fab28-14-8 complex;

and (D) Superposition of H2-D^d from 8TQ9 as compared with the pMHC from the AF-M best_model for H2-D^d from the FabS19.8 complex.

Supplementary Fig. 3.

RMSD vs residue no. – X-ray and AF-M models

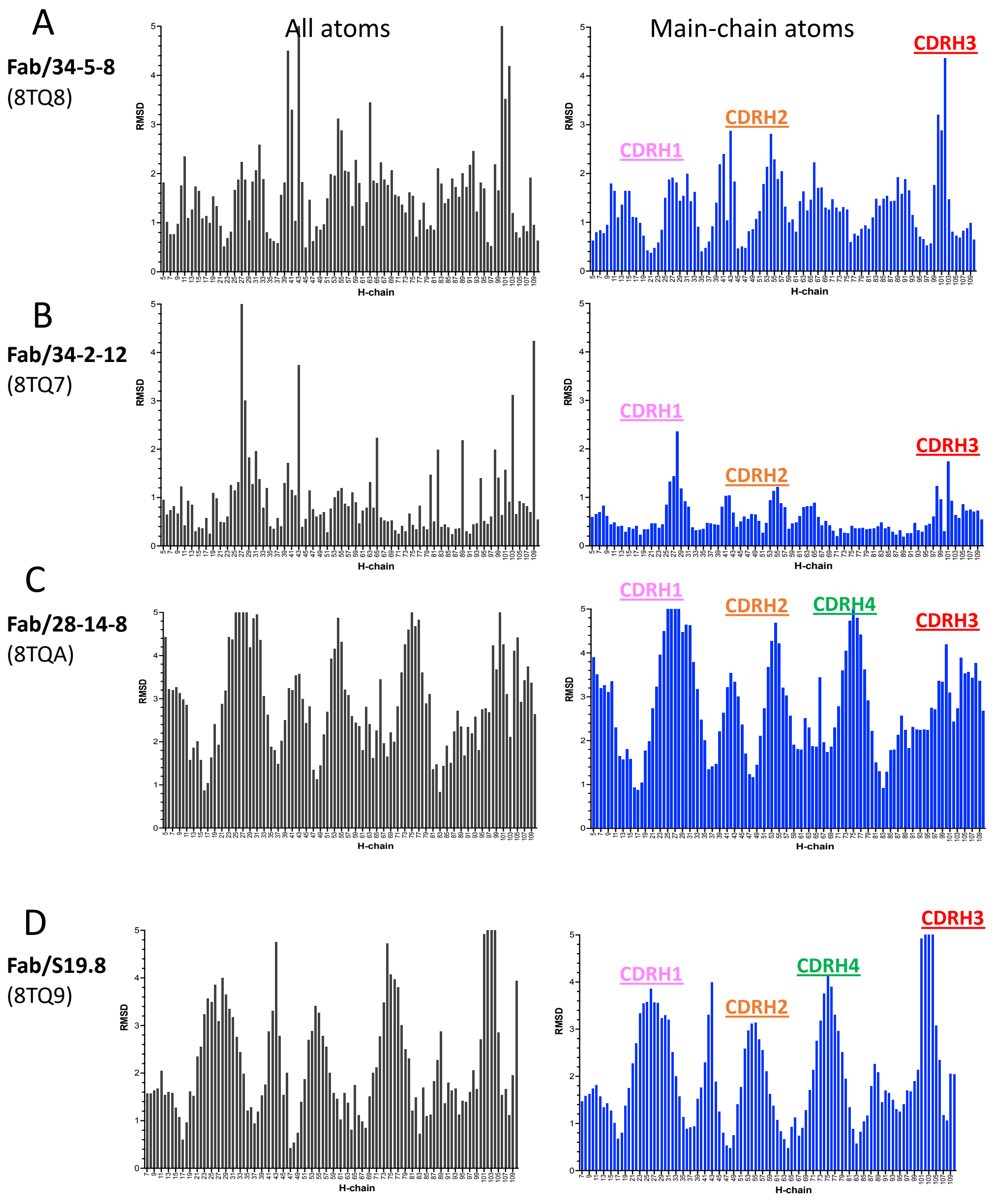

Supplementary Figure 3. Comparison of RMSD vs residue number reveals major differences in CDR loops. RMSD values determined for all atoms (left) and main-chain atoms (right) for the indicated four mAb/MHC complexes as determined crystallographically vs by AF-M.

Supplementary Figure 4.

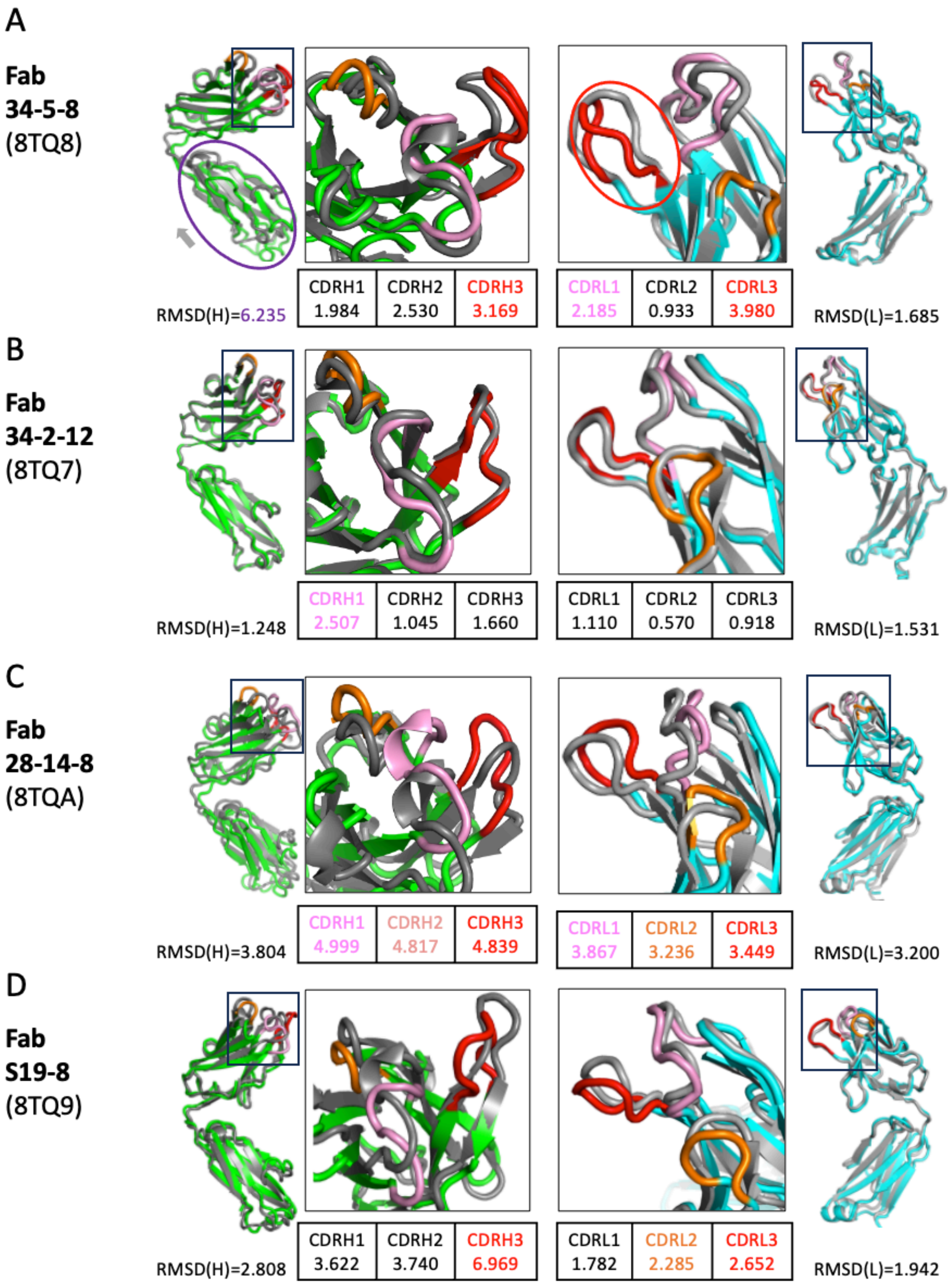

CDR1 pink, CDR2 orange, CDR3 red

Supplementary Figure 4. Illustration of major differences in conformation of CDR loops of H chains (left) and L chain (right). H or L chain comparing X-ray to AF-M structures. Superpositions of H or L chains were accomplished in PyMOL and RMSD of CDR loops were calculated as shown in illustrations or the accompanying tables.

Supplementary Fig. 5.

A

H-Chain

```
Fab3458-H       ------EESGPELGKPGASVKMSCTASGYAFTSYVMHWVMQKPGQGLEWIGYFNPYNDAG  58
Fab34212-H      ---VQLQQSGPELVKPGASVKISCQSSGYTISNSWMNWVKQRPGKGLEWIGRIYPGDGDT  58
Fab2814S-H      ------QQSGAELAKPGASVKMSCKASGYNFTTYWMHWVRQRPGQGLEWIGYINPSSGYT  58
FabS19-H        ---VQLQQSGPVLVKPGASVKMSCKASGYTFTDHYMNWVKQSPGKSLEWIGGINPYNGGI  58

Fab3458-H       KYNAKFKGKATLTSDKSSNTAYMELSSLTSEDSTVYYCARA--Y-FSKGPFAYWGQGTLV  115
Fab34212-H      HYNGKFKAKATLTADRSSSTAYMHLSSLTSEDSAIYFCARSTA-----AWFPYWGRGTLV  113
Fab2814S-H      EFNQKFKGKATLTADKSSNTAYIQLTSLTSDDSAIYYCARSF------YYFDYWGQGTTL  112
FabS19-H        SYNQKFKGMATLTADKSSSTAYMELNSLTSEDSAVYYCVRRIGY-YPSYFFDYWGQGTTL  116
```

B

L-Chain

```
Fab3458-L       ----TQTPASLAVSLGQRATISCRASESVDRHGNSFMHWYQQKPGQPPKLLIYRASNLDS  60
Fab34212-L      -TTVTQTPKFMSTSVGDRVSVTCTASQNV----DTNVAWYQQKPGQSPKPLIFSASSRYT  56
Fab2814S-L      ----TQTPSSLSASLGDRVTISCRASQDI----SNYLNWYQQKPDGPVKLLIYYTSRLHS  56
FabS19-L        -TTVTQSPASLSVATGEKVTIRCITSTDI----DDNMNWYQQKPGEPPKLLISEGNTLRP  56

Fab3458-L       GIPARFSGSGSRTDFTLTINPVEADDVATYYCQQSNEDPPWTFGGGTKLEIKRADAAPTV  120
Fab34212-L      GIPDRFGGSGSGTDFALTISNVQSEDLAEYFCQQYH-SFPYTFGGGTRLEIKRADAAPTV  115
Fab2814S-L      GVPSRFSGSGSGTDYSLTISNLEQEDIATYFCQQGN-TFPWTFGGGTKLEIKRADAAPTV  115
FabS19-L        GVPSRFSSSGFGTDFVFTIENTLSEDFADYYCLQSD-DMPLTFGAGTKLELKRADAAPTV  115
```

C

8TQ8

CDR	34-5-8
CDRH1	S25-V33
CDRH2	N52-D56
CDRH3	R98-Y108
CDRL1	S26-F36
CDRL2	R54-L58
CDRL3	Q94-W101

8TQ7

CDR	34-2-12
CDRH1	S25-M33
CDRH2	Y52-D57
CDRH3	R98-Y106
CDRL1	S26-V33
CDRL2	F49-S53
CDRL3	Q90-Y96

8TQA

CDR	28-14-8
CDRH1	S25-W33
CDRH2	N52-G56
CDRH3	R98-Y105
CDRL1	A25-L33
CDRL2	Y49-R53
CDRL3	Q90-W96

8TQ9

CDR	S19.8
CDRH1	S25-Y33
CDRH2	N52-G56
CDRH3	R99-F107
CDRL1	T25-N32
CDRL2	S49-T53
CDRL3	Q90-L96

Supplementary Figure 5. Fab Sequence Alignments and CDR ranges. Amino acid sequences of the indicated Fab H and L chains, as deduced from their respective nucleotide sequences and confirmed structurally were aligned with Clustal Omega https://www.ebi.ac.uk/seqdb/confluence/display/JDSAT/Clustal+Omega+Help+and+Documentation (*91*). These are shown for are shown for the V_H (A) and V_L (B) regions of the indicated antibodies. Dashes indicate amino acid residues that were not observed in electron density maps. Color highlights indicate structurally defined loops corresponding to the CDRs tabulated in panel C.

Fab	Chains	MHC Residue (chain A)	Ab Residue (H)	Distance (Å)
**34-5-8**	AH	GLU104	SER102	3.18
	AH	ASP106	ASN52	2.41
	AH	GLY107	THR30	3.71
	AH	ARG108	SER31	3.43
	AH	LEU109	SER31	2.63
	AH	LEU110	PHE101	3.30
	AH	ARG111	PHE101	4.74
	AH	GLU128	PHE101	3.41
	AH	ASP129	TYR100	3.34
	AH	LEU130	TYR32	3.64
	AH	LYS131	TYR100	3.38
	AH	GLU154	SER1	4.95
	AH	ARG157	GLY26	4.27
	AH	GLU161	SER31	2.57
	AH	GLY162	ALA28	4.27
	AH	VAL165	SER31	3.92
	AH	ARG169	TYR54	3.33
		**MHC Residue (chain A)**	**Ab Residue (L)**	
	AL	ASP106	GLU97	4.90
	AL	ARG108	GLU97	3.10
	AL	ASN127	TYR53	4.37
	AL	GLU128	ARG54	2.76
	AL	ASP129	SER60	4.03
	AL	THR132	SER60	3.27

Fab	Chains	MHC Residue (chain A)	Ab Residue (H)	Distance (Å)
**34-2-12**	AH	ARG194	THR96	4.92
	AH	GLU196	TRP99	2.57
	AH	GLY197	ALA97	3.29
	AH	ASP198	THR96	2.81
	AH	LEU219	ARG70	2.83
	AH	ASN220	ARG70	3.41
	AH	GLY221	ARG70	4.47
	AH	GLU222	THR24	2.38
	AH	LEU224	ASN27	4.20
	AH	GLN226	GLY22	3.29
	AH	GLU227	ASN27	2.84
	AH	VAL247	ASN27	4.18
	AH	VAL248	ASN27	2.72
	AH	VAL249	ASN27	3.51
	AH	PRO250	THR96	3.24
	AH	LEU251	THR96	4.28
	AH	LYS253	ASP51	2.62
	AH	LYS256	ASP51	3.73
	AH	TYR257	ASN27	2.29
		**MHC Residue (chain A)**	**Ab Residue (L)**	
	AL	PRO195	PHE45	3.82
	AL	GLU196	PHE45	3.66

Fab	Chains	MHC Residue (chain A)	Ab Residue (H)	Distance (Å)
**28-14-8**	AH	ARG111	LYS19	4.79
	AH	GLU128	LYS19	3.86
	AH	ASP212	TYR57	3.22
	AH	ILE213	ASN52	4.79
	AH	THR214	TRP33	3.26
	AH	THR216	PHE100	3.78
	AH	GLY221	TYR101	2.64
	AH	GLU221	TYR102	4.11
	AH	GLU223	TYR102	3.16
	AH	THR225	THR31	3.27
	AH	GLN226	TYR32	3.21
	AH	ARG260	TYR101	4.78
	AH	TYR262	TRP33	3.47

	AH	GLU264	TYR57	3.13
		**MHC Residue (chain B)**	**Ab Residue (H)**	**Distance (Å)**
	BH	ASP59	LYS74	4.87
		**MHC Residue (chain A)**	**Ab Residue (L)**	**Distance (Å)**
	AL	GLN218	TYR32	4.82
	AL	LEU219	ARG53	3.14
	AL	ASN220	ARG53	3.09
	AL	GLY221	TYR49	3.22
	AL	GLU222	TYR49	3.79
	AL	ARG260	GLY91	3.04
	AL	TYR262	PHE94	3.80
	AL	PRO267	THR93	3.57
	AL	GLU268	ASN92	3.68
	AL	PRO269	ASN92	3.16
	AL	THR271	TYR32	3.12
	AL	PHE17	THR49	3.96

Fab	Chains	MHC Residue (chain A)	Ab Residue (H)	Distance (Å)
**S19.8**	AH	SER13	TYR100	4.67
	AH	ARG14	TYR100	3.79
	AH	PRO15	TYR100	4.14
	AH	PHE17	TYR100	4.16
	AH	GLY91	TYR99	3.87
	AH	SER92	TYR99	3.62
		**MHC Residue (chain B)**	**Ab Residue (H)**	
	BH	TYR-1	ASP31	3.32
	BH	ALA0	ASP31	3.42
	BH	ILE1	TYR54	3.64
	BH	GLN2	TYR54	3.11
	BH	THR4	ASN55	4.94
	BH	PRO32	TYR102	4.40
	BH	HIS34	TYR102	3.48
	BH	GLU36	PRO104	3.91
	BH	VAL82	TYR106	4.90
	BH	LYS83	PRO104	3.24
	BH	HIS84	PRO104	4.01
	BH	ALA85	TYR102	3.49
	BH	SER86	TYR33	3.66
	BH	MET87	ARG99	2.92
	BH	ALA88	TYR33	2.96
	BH	GLU89	TYR106	3.36
	BH	PRO90	TYR106	3.25
		**MHC Residue (chain A)**	**Ab Residue (L)**	
	AL	PHE17	THR49	3.976
		**MHC Residue (chain B)**	**Ab Residue (L)**	
	BL	GLU36	ASN28	4.66
	BL	GLN38	ASP26	3.32
	BL	LYS45	ASP26	2.40
	BL	ARG81	ASP89	3.14
	BL	LYS83	SER87	3.74
	BL	ALA88	MET90	3.75
	BL	GLU89	MET90	3.54
	BL	PRO90	ASP88	3.74

Supplementary Table 1. Contacts between the indicated Fab and H2-D^d (for 34-5-8, 34-2-12, and S19.8) or H2-D^b (for 28-14-8). Contacting residues with distances $\leq$ 5 Å were evaluated as described in Methods. "A" refers to the indicated MHC-I heavy chain, "B" to indicated MHC-I L chain (b_2m), "H" and "L" to Fab heavy and light chains respectively.

Complex	PDB	Chains	BSA-H	BSA-L	BSA-(H+L)
Fab34-5-8/D^d_A	8TQ8	A (H,L)	714	211	925
Fab34-5-8/D^d_B	8TQ8	B (H,L)	0	0	0
Fab34-2-12/D^d_A	8TQ7	A (H,L)	679	78	757
Fab34-2-12/D^d_B	8TQ7	B (H,L)	0	0	0
Fab28-14-8/D^b_A	8TQA	A (H,L)	503	349	852
Fab28-14-8/D^b_B	8TQA	B (H,L)	11	0	11
FabS19.8/D^d_A	8TQ9	A (H,L)	139	52	191
FabS19.8/D^d_B	8TQ9	B (H,L)	663	243	906

Supplementary Table 2. Buried surface area (BSA) at interface between Fab and pMHC. Buried surface areas for each of the indicated interfaces were calculated with PDBePISA (*89*). Values are in Å^2.

**Fab**	X-ray Model (PDB)	AF-M Model	X-ray Elbow Angle (°)	AF-M Elbow Angle (°)	**Δ Elbow Angle (°)**	Limit-l	Limit-h
**34-5-8**	8TQ8	8TQ8-AF	150	136	**14.0**	113	119
**34-2-12**	8TQ7	8TQ7-AF	135	136	**1.0**	109	117
**28-14-8**	8TQA	8TQAAF	173	145	**28.0**	107	116
**S19.8**	8TQ9	8TQ9AF	152	168	**16.0**	107	116

Supplementary Table 3. Comparison of elbow angles of X-ray and AF-M structural models. "Limit_l" and "limit_h" indicate the residues defining the ends of variable domain of the light chain and the heavy chain respectively. The measurement of elbow angle is calculated by using the Python Script of "elbow_angle.py" (*72*) imported to PyMol.

**Molecular Complex**	**PDB for comparison**	**DockQ score**	**Evaluation**
H2-D^d/β_2m	pH2-D^d to β_2m from 8TQ8 (complex with 34-5-8)	0.448	Acceptable
H2-D^d/β_2m	pH2-D^d to β_2m from 8TQ7 (complex with 34-2-12)	0.850	High
H2-D^b/β_2m	pH2-D^d to β_2m from 8TQA (complex with 28-14-8)	0.319	Acceptable
H2-D^d/β_2m	pH2-D^d to β_2m from 8TQ9 (complex with S19.8)	0.474	Acceptable
Fab34-5-8 H to L	Fab H to L from 8TQ8	0.525	Acceptable
Fab34-2-12 H to L	Fab H to L from 8TQ7	0.879	High
Fab28-14-8 H to L	Fab H to L from 8TQA	0.606	Medium
FabS19.8 H to L	Fab H to L from 8TQ9	0.718	Medium
Fab34-5-8/H2-D^d	Complete Fab to pMHC of 8TQ8	0.027	Incorrect
Fab34-2-12/H2-D^d	Complete Fab to pMHC of 8TQ7	0.007	Incorrect
Fab28-14-8/H2-D^b	Complete Fab to pMHC of 8TQA	0.015	Incorrect
FabS19.8/H2-D^d	Complete Fab to pMHC of 8TQ9	0.023	Incorrect

Supplementary Table 4. Comparison of docking of X-ray and AF-M complexes using DockQ. To compare the veracity of pMHC/b_2m complexes, Fab H/L complexes, and docked Fab/pMHC-I complexes, the X-ray and AF-M coordinates were compared using DockQ (*56*). For the indicated complexes a value of DockQ was calculated. As suggested, DockQ scores were considered: incorrect, 0<0.2; acceptable, 0.2<0.5; medium, 0.5<0.8; and high, 0.8<1.0.
